# Comparison of prevalence and risk factors of somatization between Chinese health care workers and non-health care workers during COVID-19 outbreak

**DOI:** 10.1186/s12888-021-03294-z

**Published:** 2021-05-31

**Authors:** Xiuli Song, Yongjie Zhou, Wenwang Rao, Xiangyang Zhang

**Affiliations:** 1 Clinical Psychology, Yantai Affiliated Hospital of Binzhou Medical University, Yantai, China; 2 Shenzhen Kangning Hospital, Shenzhen, China; 3 Unit of psychiatry, Faculty of Health Sciences, University of Macau, Macau, SAR China; 4 CAS Key Laboratory of Mental Health, Institute of Psychology, Chinese Academy of Sciences, Beijing, China

**Keywords:** COVID-19, Health care workers, Somatization, Risk factors

## Abstract

**Background:**

This study aimed to compare prevalence and risk factors of somatization (SOM) between health care workers and non-health care workers during COVID-19 outbreak in China.

**Methods:**

From 14 February to 29 March 2020, an online survey was performed in both 605 health care workers and 1151 non-health care workers. Based on the somatization dimension score of the Symptom Checklist-90, participants were divided into non-SOM group and SOM group.

**Results:**

Health care workers had higher prevalence rate of SOM (*p* < 0.001) than non-health care workers, with an OR of 1.70 (95% CI, 1.22–2.36, *p* = 0.002). Multiple logistic regression analysis revealed that in non-health care workers, the risk factors of SOM included other ethnicities, insomnia, and suicide, while in health care workers, the risk factors included working 6–8 h per day, and working ≥10 h per day during COVID-19 outbreak.

**Conclusions:**

Our research suggests that both non-health care workers and health care workers have a relatively high prevalence of somatization. However, the related factors for somatization in both groups are significantly different, showing that medical service-related factors are associated with somatization in health care workers, while demographic and clinical factors are associated with somatization in non-health care workers.

## Background

The coronavirus disease 2019 (COVID-19) epidemic first occurred in Wuhan city, Hubei province, China, and then spread rapidly nationwide from December 2019 up to date [1]. During the COVID-19 epidemic, a total of 7731 confirmed cases and 170 deaths were reported by 30 January. As the COVID-19 epidemic spread rapidly to all provinces in China within a month, the World Health Organization (WHO) announced the COVID-19 outbreak as a Public Health Emergency of International Concern (PHEIC) (World Health Organization, 2020a) on January 30, 2020 [2]. From then on, the COVID-19 epidemic has been spreading fast all over the world. According to Dr. Tedros, Director-General of WHO, the threat of a global pandemic caused by the coronavirus is now very real. On March 11, 2020, the WHO declared COVID-19 as a pandemic [2].

To efficiently control the COVID-19 outbreak, the Chinese government launched the Public Health Emergency Response (level I) in mainland China on January 29 [3], which means that some practical measures have been implemented, including partial blockades in most cities, community lockdown, cancelation of activities, suspension of most means of transportion, and prohibition of unnecessary gatherings [2, 4, 5]. These measures are aimed at reducing the probability of transmission between infected and uninfected persons [6]. However, the implementation of the above-mentioned measures may also bring some problems, which will have an impact on social norms, interpersonal relationship, the economy and the psychological wellbeing of the population [5]. Previous studies have shown that distress is inevitable if people who suffer from restrictions, loss of daily routines, and lack of interpersonal communication with others can frequently have feelings of boredom, headache, frustration, loneliness and isolation from the world [7–9]. The distress may be exacerbated when people are unable to participate in daily activities for a long time [10].

In addition to isolation, people’s mental health is also affected by the rapid spread of the COVID-19 disease, its severity, increased incidences and mortality, lack of effective treatment and vaccines, and the availability of basic resources, such as hand sanitizers, facial masks and digital thermometers. These problems can lead to mental disorders, including depression and anxiety disorders, insomnia and posttraumatic stress disorder (PTSD) [11], which in turn may cause more serious harm than the COVID-19 epidemic itself [3]. Some studies have shown that infectious diseases can bring psychological changes not only to health care workers but also to non-health care workers [12, 13], suggesting that COIVD-19 can cause psychological changes [1, 2, 14–16]. For example, one study conducted by Zhang et al. showed that during the outbreak of COVID-19 in China, more than 33% of medical staffs developed symptoms of insomnia from January 29 to February 3 [17]. Another study performed by Lai et al. in China indicated that from January 29 to February 3, the prevalence rates of distress symptoms, depression, anxiety and insomnia were 71.5, 50.4, 44.6, 34.0%, respectively [18]. Tian and his colleagues found that from January 31 to February 2, more than 70% of participants had moderate to severe psychological symptoms, especially obsessive compulsive disorder, phobic anxiety, interpersonal sensitivity and psychiatric disorders in the Chinese general population [2]. Chew et al. found that physical symptoms were associated with higher average scores calculated by the Impact of Events Scale-Revised (IES-R) and Depression Anxiety Stress Scales (DASS-21) for healthcare workers in Singapore and India from February 19 to April 17, 2020 [16].

To date, there have been few studies on the comparison of psychological symptoms between non-health care workers and health care workers during the COVID-19 outbreak in China and other countries. For instance, Tian et al. found that there was a significant difference in the somatization score of SCL-90 between health care workers and general population (1.81 ± 0.69 vs 1.37 ± 0.48, *p* = 0.001) [2]. Chew et al. reported that there was a significant association between the risk of physical symptoms and psychological distress among health care workers during the COVID-19 epidemic [16]. Therefore, the main purposes of this study were to (1) compare the differences in demographic and clinical data between non-health care workers and health care workers; and (2) to explore the risk factors of somatization severity shown on SCL-90 scale between health care workers and non-health care workers.

## Materials and methods

### Study design and participants

This is an online epidemiological survey using a self-administered questionnaire during the COVID-19 epidemic to minimize face-to-face interaction. Using a cross-sectional design, all anonymous online questionnaires were distributed presented in the form of posters through “Wenjuanxing” Survey Platform (Ranxing Technology, China) in China from February 14 to March 29, 2020, which was forwarded through Wechat and other channels in the form of posters. The recruited participants logged in by through scanning the QR code and filled in the questionnaire. Totally, this study recruited 1756 participants including 1151 non-health care workers and 605 health care workers. The collected socio-demographic data included: gender, age, height, weight, ethnicity, marital status, education level, city of residence, occupation (physician or nurse), daily working hours, annual family income, history of somatic diseases, experience of SARS epidemic), and infection with COVID-19 in relatives and friends.

In this study, the inclusion criteria was that the participants must be an adult Chinese citizen between the ages of 18 and 70 years. All participants were in good physical health. Exclusion criteria included those subjects with medical/surgical problems, organic brain diseases, or severe somatic diseases. Subjects who met the exclusion criteria were ruled out by self-reporting through the questions on online questionnaires.

This study protocol was reviewed and approved by the Institute of Psychology, Chinese Academy of Sciences. Ethical approval was conducted in accordance with the latest version of the Helsinki Declaration (line 96–98). All participants received an electronic informed consent form and then signed the form to participate in the study.

### Assessment

Demographic data, self-designed questionnaire related to the COVID-19 outbreak, and the SOM dimension of SCL-90 scale, the Insomnia Severity Index (ISI) scale, and suicide module of Mini International Neuropsychiatric Interview (MINI) were obtained through the “Wenjuanxing” Survey Platform.

SCL-90 was used to measure psychological distress and psychopathological symptoms [19]. The 90-item self-reported symptom survey is categorized into nine dimensions: Somatization (SOM), Obsessive-Compulsive (OC), Interpersonal Sensitivity (IS), Depression (DEP), Anxiety (ANX), Hostility (HOS), Phobic Anxiety (PHOB), Paranoid Ideation (PAR), and Psychoticism (PSY). We selected the SOM dimension (12 items) of the SCL-90 to assess the severity of physical discomfort. Each item is scored on a 1–5 scale, and the total score ranges from 12 to 60. The total score is divided into different degrees of somatic discomfort symptoms: no somatic discomfort (< 24, i.e., non-SOM group), as well as minimal, moderate and severe somatic discomfort (≥ 24, i.e., SOM group).

The Insomnia Severity Index (ISI) was performed to assess the severity of insomnia symptoms [20]. Each item is graded on a scale of 0–4, and the total score of the 7–item ISI ranges from 0 to 28. The total score is categorized into four different groups: no insomnia (0–7), mild (8–14), moderate (15–21), and severe (22–28).

Mini International Neuropsychiatric Interview (MINI) was designed as a brief structured interview for major Axis I psychiatric disorders in DSM-IV and ICD-10 [21]. We selected the suicide module (7 items) of the MINI to assess the severity of suicide symptoms. The total score is classified into: no (0), mild (1–5), moderate (6–9), and severe (≥10).

### Statistical analysis

Data were analyzed using SPSS statistical software for Windows (version 22.0., IBM Corp.). The categorized variables between the two groups were analyzed by chi-square test. Kolmogorov-Smirnov single sample test was used to assess the normality of continuous variables. For the data of normal distribution, the independent Student’s t-test was used to compare the differences between two groups. For the data of non-normal distribution, the median and the interquartile ranges (IQRs) were presented and the Wilcoxon test (Mann–Whitney test) was used to compare the differences between two groups. Demographic data and clinical symptoms were analyzed with 2 × 2 ANOVA representing the between factors of group (non-health care workers vs. health care workers) and diagnose (non-SOM group vs. SOM group). Finally, multiple logistic regression analysis with the forward stepwise method was carried out to examine potential risk factors of SOM in different groups. *P*-value < 0.05 with 2-tailed tests was regarded as statistical significance.

## Results

### Prevalence of somatization between non-health care workers and health care workers

A total of 1756 participants completed the survey, including 1151 (65.55%) non-health care workers and 605 (34.45%) health care workers. The prevalence rate of SOM in health care workers was 9.59%, which was significantly higher than that in non-health care workers (5.45%), with an OR of 1.70 (95% confidence interval: 1.22–2.36; χ^2^ = 9.80, df = 1, *p* = 0.002). After controlling for the sociodemographic confounders, such as gender, age, ethnicity, education, marital status, living situation and BMI, logistic regression analysis showed that there was still a significant difference, with an adjusted OR of 1.66 (95% CI: 1.15–2.39; χ^2^ = 7.26, *p* = 0.007). Further, there were significant differences between non-health care workers and health care workers in terms of sex, age, body mass index (BMI), ethnicity, marital status, education level, living status, relatives and friends infected with COVID-19, experience SARS personally, income level, economic loss, medical disease, somatization, insomnia, and drinking (all *p* < 0.05) (Table 1).

**Table 1:** Demographic data and clinical symptoms between non-health care workers and health care workers

Variables	Non-health care workers (*n* = 1151)	Health care workers (*n* = 605)	Z/χ2	*p*
Sex, n (%)				
Men	356(30.93)	114(18.84)	29.56	< 0.001
Women	795(69.07)	491(81.16)		
Age (years), Median (IQR)	22(21–37)	35(30–41)	−15.81	< 0.001
BMI (kg/m^2^), Median (IQR)	21.51(19.72–24.16)	22.03(20.22–24.35)	−2.57	0.01
Ethnicity, n (%)				
Han	1092(94.87)	558(92.23)	4.88	0.03
Others	59(5.13)	47(7.77)		
Marital status, n (%)				
Single	689(59.86)	122(20.17)	260.30	< 0.001
Married or cohabiting	413(35.88)	456(75.37)		
Divorced, separated or widowed	49(4.26)	27(4.46)		
Education level, n (%)				
High school or below	148(12.86)	14(2.31)	110.32	< 0.001
Junior college and Bachelor’s degree	897(77.93)	445(73.55)		
Master’s degree or above	106(9.21)	146(24.13)		
Occupation, n (%)				
Student	586(50.91)	NA		
Professional	339(29.45)	NA		
Teacher	57(4.95)	NA		
Others	189(16.42)	NA		
Doctor	NA	208(34.38)		
Nurse	NA	333(55.04)		
Medical technician	NA	64(10.58)		
Living situation, n (%)				
Wuhan	17(1.48)	28(4.63)	37.98	< 0.001
Hubei province outside Wuhan	14(1.22)	29(4.79)		
Outside Hubei province	1120(97.31)	548(90.58)		
Length of service (years), n (%)				
≤ 5	NA	110(18.18)		
6–10	NA	162(26.78)		
≥ 10	NA	194(32.07)		
≥ 20	NA	139(22.98)		
Working hours per day during COVID-19 outbreak, n (%)				
4–6	NA	40(6.61)		
6–8	NA	243(40.17)		
8–10	NA	268(44.30)		
≥ 10	NA	54(8.93)		
Relatives and friends infected with coronavirus, n (%)				
No	1143(99.30)	592(97.85)	7.09	0.01
Yes	8(0.70)	13(2.15)		
Experience SARS personally, n (%)				
No	594(51.61)	343(56.69)	4.12	0.04
Yes	557(48.39)	262(43.31)		
Income (ten thousand), n (%)				
≤ 8	488(42.40)	105(17.36)	111.35	< 0.001
8–30	539(46.83)	402(66.45)		
≥ 30	124(10.77)	98(16.20)		
Income (ten thousand, Chinese Yuan)	18.2 ± 17.5	24.1 ± 18.7	4.99	< 0.001
Economic loss (ten thousand), n (%)				
≤ 3	791(68.72)	323(53.39)	56.79	< 0.001
3–10	82(7.12)	89(14.71)		
≥ 10	82(7.12)	83(13.72)		
Unknown	202(17.55)	110(18.18)		
Medical illness, n (%)				
No	1002(87.05)	468(77.36)	27.36	< 0.001
Yes	149(12.95)	137(22.64)		
Somatization, n (%)				
No (< 24)	1108(96.26)	547(90.41)	11.12	< 0.001
Yes (≥24)	63(5.45)	58(9.59)		
Insomnia, Median (IQR)	3(0–7)	5(1–9)	−4.44	< 0.001
Suicide (mean ± SD)	0.19 ± 0.61	0.22 ± 0.63	−1.05	0.30
Smoking, n (%)				
No	1032(89.66)	550(90.91)	0.94	0.62
Yes	82(7.12)	40(6.61)		
smoking cessation	37(3.21)	15(2.48)		
Drinking, n (%)				
No	884(76.80)	460(76.03)	7.21	0.03
Yes	212(18.42)	130(21.49)		
Abstinence	55(4.78)	15(2.48)		

### Comparison of SCL-90 SOM dimension subscale between non-health care workers and health care workers

Health care workers had higher SOM total score (*p* < 0.001) than non-health care workers. Each item score of SOM was significantly higher in health care workers than that in non-health care workers (*p* < 0.001 ~ *p* < 0.05) (Table 2).

**Table 2:** Comparison of SCL-90 SOM dimension subscale between non-health care workers and health care workers

Variables	Non-health care workers (n = 1151)	health care workers (n = 605)	
total scores	14.0 (12.0–17.0)	16.0 (14.0–20.0)	< 0.001
Headaches	1.0 (1.0–2.0)	2.0 (1.0–2.0)	< 0.001
Faintness or dizziness	1.0 (1.0–1.0)	1.0 (1.0–2.0)	< 0.001
Pains in heart or chest	1.0 (1.0–1.0)	1.0 (1.0–1.0)	0.003
Pains in lower back	1.0 (1.0–2.0)	2.0 (1.0–2.0)	< 0.001
Nausea or upset stomach	1.0 (1.0–2.0)	1.0 (1.0–2.0)	< 0.001
Soreness of your muscles	1.0 (1.0–2.0)	2.0 (1.0–2.0)	< 0.001
Trouble getting your breath	1.0 (1.0–1.0)	1.0 (1.0–1.0)	< 0.001
Hot or cold spells	1.0 (1.0–1.0)	1.0 (1.0–1.0)	< 0.001
Numbness or tingling in parts of your body	1.0 (1.0–1.0)	1.0 (1.0–1.0)	0.02
A lump in your throat	1.0 (1.0–1.0)	1.0 (1.0–1.5)	0.046
Feeling weak in parts of your body	1.0 (1.0–2.0)	1.0 (1.0–2.0)	< 0.001
Heavy feelings in your arms or legs	1.0 (1.0–1.0)	1.0 (1.0–2.0)	< 0.001

### Comparison of demographic data and clinical symptoms by group and diagnose

As shown in Table 3, two-way ANOVA showed that there were significant effects of group on age, marital status, education level, living status, income level, economic loss, insomnia, and suicide (all *p* < 0.05). There were significant effects of diagnose on age, experience SARS personally, medical illness, insomnia, suicide, and drinking (all *p* < 0.05). Also, there were significant group ×diagnose effects on sex, medical illness, insomnia, and suicide (*p* < 0.05).

**Table 3:** Comparison of demographic data and clinical symptoms by group and diagnose

variables	Non-health care workers		health care workers		Group		Diagnose		Group×Diagnose	
	Non-SOM group	SOM group	Non-SOM group	SOM group	F	*p*	F	*p*	F	*p*
	(*n* = 1065)	(*n* = 86)	(*n* = 532)	(*n* = 73)						
Sex, n (%)					2.94	0.09	3.44	0.06	4.24	0.04
Men	330(30.99)	26(30.23)	91(17.11)	23(31.51)						
Women	735(69.01)	60(69.77)	441(82.89)	50(68.49)						
Age (years)	28.43 ± 0.30	31.17 ± 1.04	35.67 ± 0.42	36.53 ± 1.13	60.17	< 0.001	4.93	0.03	1.34	0.25
BMI (kg/m^2^)	22.20 ± 0.11	22.41 ± 0.39	22.61 ± 0.16	22.94 ± 0.42	2.41	0.12	0.83	0.36	0.03	0.85
Ethnicity, n (%)					0.02	0.90	0.23	0.63	3.18	0.08
Han	1014(95.21)	78(90.70)	489(91.92)	69(94.52)						
Others	51(4.79)	8(9.30)	43(8.08)	4(5.48)						
Marital status, n (%)					68.93	< 0.001	2.8	0.10	0.24	0.63
Single	648(60.85)	41(47.67)	111(20.86)	11(15.07)						
Married or cohabiting	369(34.65)	44(51.16)	397(74.62)	59(80.82)						
Divorced, separated or widowed	48(4.51)	1(1.16)	24(4.51)	3(4.11)						
Education level, n (%)					47.28	< 0.001	0.64	0.42	0.19	0.66
High school or below	134(12.58)	14(16.28)	13(2.44)	1(1.37)						
Junior college and Bachelor’s degree	832(78.12)	65(75.58)	389(73.12)	56(76.71)						
Master’s degree or above	99(9.30)	7(8.14)	13(2.44)	1(1.37)						
Living situation, n (%)					7.94	0.005	3.91	0.05	0.73	0.39
Wuhan	17(1.60)	0(0.00)	27(5.08)	1(1.37)						
Hubei province outside Wuhan	13(1.22)	1(1.16)	26(4.89)	3(4.11)						
Outside Hubei province	1035(97.18)	85(98.84)	479(90.04)	69(94.52)						
Relatives and friends infected with coronavirus, n (%)					2.49	0.12	0.81	0.37	0.01	0.94
No	1057(99.25)	86(100)	520(97.74)	72(98.63)						
Yes	8(0.75)	0(0.00)	12(2.26)	1(1.37)						
Experience SARS personally, n (%)					1.28	0.26	4.62	0.03	0.05	0.82
No	556(52.21)	38(44.19)	308(57.89)	35(47.95)						
Yes	509(47.79)	48(55.81)	224(42.11)	38(52.05)						
Income (ten thousand), n (%)					42.32	< 0.001	1.68	0.20	1.02	0.31
≤ 8	448(42.07)	40(46.51)	97(18.23)	8(10.96)						
8–30	507(47.61)	32(37.21)	352(66.17)	50(68.49)						
≥ 30	110(10.33)	14(16.28)	83(15.60)	15(20.55)						
Economic loss (ten thousand), n (%)					9.9	0.002	0.21	0.65	0.77	0.38
≤ 3	734(68.92)	57(66.28)	286(53.76)	37(50.68)						
3–10	72(6.76)	10(11.63)	79(14.85)	10(13.70)						
≥ 10	74(6.95)	8(9.30)	74(13.91)	9(12.33)						
Unknown	185(17.37)	11(12.79)	93(17.48)	17(23.29)						
Medical illness, n (%)					0.07	0.80	4.85	0.03	12.19	< 0.001
No	124(11.64)	25(29.07)	123(23.12)	14(19.18)						
Yes	941(88.36)	61(70.93)	409(76.88)	59(80.82)						
Insomnia	4.52 ± 0.17	11.15 ± 0.60	6.22 ± 0.24	6.44 ± 0.65	10.31	0.001	53.35	< 0.001	46.77	< 0.001
Suicide	0.71 ± 0.12	4.41 ± 0.43	1.07 ± 0.17	0.81 ± 0.46	23.82	< 0.001	26.92	< 0.001	35.65	< 0.001
Smoking, n (%)					0.12	0.73	0.67	0.42	0.11	0.74
No	955(89.67)	77(89.53)	486(91.35)	64(87.67)						
Yes	77(7.23)	5(5.81)	33(6.20)	7(9.59)						
smoking cessation	33(3.10)	4(4.65)	13(2.44)	2(2.74)						
Drinking, n (%)					1.08	0.30	5.66	0.02	0.53	0.47
No	825(77.46)	59(68.60)	409(76.88)	51(69.86)						
Yes	193(18.12)	19(22.09)	110(20.68)	20(27.40)						
Abstinence	47(4.41)	8(9.30)	13(2.44)	2(2.73)						

### Demographic data and clinical symptoms between non-SOM and SOM groups of non-health care workers

All non-health care workers were divided into two groups: non-SOM group (SOM total score < 24) and SOM group (SOM total score ≥ 24). There were significant differences in age, marital status, occupation, medical illness, insomnia, and suicide between two groups (Table 4). Multiple logistic regression showed that other ethnicities (non-Han Chinese) (OR = 2.45, *p* = 0.04), insomnia (OR = 1.16, *p* < 0.001), suicide (OR = 1.08, *p* < 0.001) and being single (OR = 0.52, *p* = 0.01) were associated with the SOM of non-health care workers (Table 5).

**Table 4:** Demographic data and clinical symptoms between non-SOM and SOM groups of non-health care workers

	Non-health care workers			
	Non-SOM group (n = 1065)	SOM group (n = 86)	Z/χ2	*P*
Sex, n (%)				
Men	330(30.99)	26(30.23)	0.02	0.88
Women	735(69.01)	60(69.77)		
Age (years), Median (IQR)	22(21–37)	30.50(22–38)	−2.91	< 0.001
BMI (kg/m2), Median (IQR)	21.51(19.71–24.17)	21.50(20.02–23.89)	−0.74	0.46
Ethnicity, n (%)				
Han	1014(95.21)	78(90.70)	3.33	0.07
Other	51(4.79)	8(9.30)		
Marital status, n (%)				
Single	648(60.85)	41(47.67)	10.45	0.005
Married or cohabiting	369(34.65)	44(51.16)		
Divorced, separated or widowed	48(4.51)	1(1.16)		
Education level, n (%)				
High school or below	134(12.58)	14(16.28)	1.03	0.60
Junior college and Bachelor’s degree	832(78.12)	65(75.58)		
Master’s degree or above	99(9.30)	7(8.14)		
Occupation, n (%)				
Student	539(50.61)	30(34.88)	7.92	0.048
Professional	306(28.73)	33(38.37)		
Teacher	51(4.79)	5(5.81)		
Other	169(15.87)	18(20.93)		
Living situation, n (%)				
Wuhan	17(1.60)	0(0.00)	1.40	0.50
Hubei province outside Wuhan	13(1.22)	1(1.16)		
Outside Hubei province	1035(97.18)	85(98.84)		
Relatives and friends infected with coronavirus, n (%)				
No	1057(99.25)	86(100)	0.65	0.42
Yes	8(0.75)	0(0.00)		
Experience SARS personally, n (%)				
No	556(52.21)	38(44.19)	2.05	0.15
Yes	509(47.79)	48(55.81)		
Income, n (%)				
≤ 8	448(42.07)	40(46.51)	4.82	0.09
8–30	507(47.61)	32(37.21)		
≥ 30	110(10.33)	14(16.28)		
Economic loss, n (%)				
≤ 3	734(68.92)	57(66.28)	4.33	0.23
3–10	72(6.76)	10(11.63)		
≥ 10	74(6.95)	8(9.30)		
Unknown	185(17.37)	11(12.79)		
Medical illness, n (%)				
Yes	124(11.64)	25(29.07)	21.44	< 0.001
No	941(88.36)	61(70.93)		
Insomnia, Median (IQR)	3(0–7)	12(6–15)	−8.86	< 0.001
Suicide (mean ± SD)	0.15 ± 0.53	0.76 ± 1.11	− 8.72	< 0.001
Smoking, n (%)				
No	955(89.67)	77(89.53)	0.82	0.66
Yes	77(7.23)	5(5.81)		
Smoking cessation	33(3.10)	4(4.65)		
Drinking, n (%)				
No	825(77.46)	59(68.60)	5.48	0.07
Yes	193(18.12)	19(22.09)		
Abstinence	47(4.41)	8(9.30)

**Table 5:** Multiple logistic regression analysis of SOM-related factors of non-health care workers

Variable	B	SE	*p*	OR	95%CI	
Ethnicity (ref: Han)	0.90	0.44	0.04	2.45	1.04–5.78	
Insomnia	0.15	0.02	< 0.001	1.16	1.12–1.20	
Suicide	0.07	0.02	< 0.001	1.08	1.04–1.12	
Marital status (ref: Married or cohabiting)			0.01			
Single	−0.66	0.25	0.01	0.52	0.32–0.84	
Divorced, separated or widowed	−1.99	1.05	0.06	0.14	0.02–1.08	
Constant	−6.27	1.16	< 0.001	0.002		.

### Demographic data and clinical symptoms in non-SOM and SOM groups of health care workers

All health care workers were divided into two groups: non-SOM group and SOM group. There were significant differences in sex, and working hours per day during COVID-19 outbreak between non-SOM group and SOM group of health care workers (Table 6). Further multiple logistic regression showed that women (OR = 0.46, *p* = 0.01), working 6–8 h per day during COVID-19 outbreak (OR = 14.87, *p* = 0.01), and working ≥10 h per day during COVID-19 outbreak (OR = 11.07, *p* = 0.02) were independently associated with the SOM of health care workers (Table 7).

**Table 6:** Demographic data and clinical symptoms between non-SOM and SOM groups of health care workers

	Health care workers			
	Non-SOM group (n = 532)	SOM group (n = 73)	Z/χ2	*p*
Sex, n (%)				
Men	91(17.11)	23(31.51)	8.71	0.003
Women	441(82.89)	50(68.49)		
Age (years), Median (IQR)	35(30–41)	36(30–44)	−0.93	0.35
BMI (kg/m2), Median (IQR)	22.03(20.20–24.43)	22.60(20.71–24.60)	−0.82	0.41
Ethnicity, n (%)				
Han	489(91.92)	69(94.52)	0.61	0.44
Other	43(8.08)	4(5.48)		
Marital status, n (%)				
Single	111(20.86)	11(15.07)	1.42	0.49
Married or cohabiting	397(74.62)	59(80.82)		
Divorced, separated or widowed	24(4.51)	3(4.11)		
Education level, n (%)				
High school or below	13(2.44)	1(1.37)	0.60	0.74
Junior college and Bachelor’s degree	389(73.12)	56(76.71)		
Master’s degree or above	130(24.44)	16(21.92)		
Occupation, n (%)				
Doctor	180(33.83)	28(38.36)	2.44	0.30
Nurse	292(55.89)	41(56.16)		
Medical technician	60(11.28)	4(5.48)		
Living situation, n (%)				
Wuhan	27(5.08)	1(1.37)	2.13	0.35
Hubei province outside Wuhan	26(4.89)	3(4.11)		
Outside Hubei province	479(90.04)	69(94.52)		
Length of service, n (%)				
≤ 5	99(18.61)	11(15.07)	1.65	0.65
6–10	144(27.07)	18(24.66)		
≥ 10	166(31.20)	28(38.36)		
≥ 20	123(23.12)	16(21.92)		
Working hours per day during COVID-19 outbreak, n (%)				
4–6	31(5.83)	9(12.33)	15.38	0.002
6–8	222(41.73)	21(28.77)		
8–10	226(42.48)	42(57.53)		
≥ 10	53(9.96)	1(1.37)		
Relatives and friends infected with coronavirus, n (%)				
No	520(97.74)	72(98.63)	0.24	0.63
Yes	12(2.26)	1(1.37)		
Experience SARS personally, n (%)				
No	308(57.89)	35(47.95)	2.59	0.11
Yes	224(42.11)	38(52.05)		
Income, n (%)				
≤ 8	97(18.23)	8(10.96)	2.98	0.23
8–30	352(66.17)	50(68.49)		
≥ 30	83(15.60)	15(20.55)		
Economic loss, n (%)				
≤ 3	286(53.76)	37(50.68)	1.48	0.69
3–10	79(14.85)	10(13.70)		
≥ 10	74(13.91)	9(12.33)		
Unknown	93(17.48)	17(23.29)		
Medical illness, n (%)				
Yes	123(23.12)	14(19.18)	0.57	0.45
No	409(76.88)	59(80.82)		
Insomnia, Median (IQR)	5(1–9)	5(1–7)	−0.01	0.99
Suicide (mean ± SD)	1.07 ± 2.03	0.81 ± 2.69	− 0.95	0.34
Smoking, n (%)				
No	486(91.35)	64(87.67)	1.23	0.54
Yes	33(6.20)	7(9.59)		
Smoking cessation	13(2.44)	2(2.74)		
Drinking, n (%)				
No	409(76.88)	51(69.86)	1.79	0.41
Yes	110(20.68)	20(27.40)		
Abstinence	13(2.44)	2(2.73)

**Table 7:** Multiple logistic regression analysis of SOM-related factors of health care workers

	B	SE	*p*	OR	95%CI	
Sex (ref: Men)	−0.79	0.29	0.01	0.46	0.26–0.80	
Working hours per day during COVID-19 outbreak (ref: [4–6])			0.01			
6–8	2.70	1.08	0.01	14.87	1.79–123.78	
8–10	1.78	1.04	0.09	5.91	0.77–45.26	
≥10	2.40	1.03	0.02	11.07	1.48–82.75	
Constant	−2.70	1.10	0.01	0.07		.

## Discussion

To the best of our knowledge, this was the first study to compare the somatization dimension of SCL-90 between non-medical staff and medical staff during the COVID-19 epidemic. The results showed that: (1) during the epidemic of COVID-19, the prevalence rate of somatization of medical staff was higher than non-medical staff; (2) there were significant differences in the total score of somatization and the scores of each item of somatization between non-medical staff and medical staff; (3) the daily working hours during the epidemic period of COVID-19 was the risk factor for somatization of medical staff, while the female gender was the protective factor; (4) ethnicity, singleness, insomnia and suicide were the risk factors for somatization of non-medical staff. The results of this study were of great significance to the formulation of psychological support and intervention measures for different populations during the outbreak of COVID-19.

Our findings were consistent with those in one recent study [22] showing that the prevalence rate of somatization in medical staff was higher than that in non-medical staff. Previous studies have shown that somatization refers to the transition from mental state to physical symptoms [23]. Somatic symptoms are defined as a group of physical disorders, such as digestion, appetite, sleep, or physical unhappiness or worry that are not pretending or intentional [24]. These symptoms are or are not caused by organic diseases. An early study suggests that headaches may be associated with the accumulation of adverse psychological effects or the deterioration of their pre-existing medical conditions [16]. Another study shows that the general population has a higher prevalence of depression and anxiety, and they are more likely to develop certain symptoms when experiencing the COVID-19 epidemic, such as cough, chills, dizziness, sore throat and muscle pain [14]. A large number of negative information, including the asymptomatic transmission of the virus carriers and COVID-19, often lead to adverse psychological consequences and may produce a variety of somatic symptoms [25, 26].

The real relationship between medical symptoms and psychological stress actually faces enormous challenges, especially in the current tense situation. Before giving a “non-specific” symptom diagnosis, each suspected case needs to urgently rule out any potential possibility [16]. Once the acute infection is solved, psychological support and intervention should be carried out immediately. The COVID-19 epidemic may bring psychological problems to non-health care workers and health care workers, which may turn into physical symptoms. Compared with the isolation of the general population, health care workers need to get along with patients face-to-face, working long hours and high intensity, so they are more dangerous and more prone to psychological problems. In this study, it is found that the total score of SOM and the score of each item of medical staff are higher than those of non-medical staff. Therefore, when people have somatic symptoms, they must carry out psychological intervention after excluding organic diseases.

Our study found that minor ethnicities (non-Han Chinese) were a risk factor. We speculated that there may be some possible reasons. For example, most ethnic minorities live in remote areas and have relatively poor medical conditions. According to a previous study, living in rural areas was a risk factor for somatization in the entire sample, as the population in rural areas may worry about infection due to poor medical skills and conditions [22]. COVID-19 is characterized by human-to-human transmission [27, 28], high incidence and potentially lethal [18, 29], which may enhance people’s perception of personal danger. With the increase of COVID-19’s confirmed and suspected cases, ordinary people have begun to worry about their health, family health and public health when they are quarantined at home or lost contact with the outside world. In particular, they worry about physical symptoms that may be associated with infection [7], such as cough, dizziness and fever. Further, our study found being single was a protective factor. The possible reason may be that they could not be infected with the coronavirus as long as they ensured their own personal hygiene and did not come into contact with others, when they were alone. A recent study has shown that insomnia may lead to psychological problems related to the epidemic of COVID-19 [17]. When psychological problems cannot be expressed directly, they may be expressed in the form of physical symptoms. Some studies suggest that the highly somatization group had higher suicide attempts and more individual attempts [23]. Isolation can lead to uncomfortable feelings, such as loss of freedom, loneliness from separation from love, and worry about uncertain illness. One study found that when people were quarantined during the previous outbreak, suicides followed [30]. Therefore, in our study, insomnia and suicide were associated with severe physical symptoms in non-health care workers.

Our results showed that female gender was a protective factor for health care workers. The significant differences in personality characteristics (expression and implication) between women and men can partly explain this. After a short period of training, health care workers were asked to join the front-line battle against COVID-19. Health care workers were always in contact with infected patients. Moreover, during the COVID-19 outbreak, health care workers worked continuously under negative pressure for more than 12 h and were equipped with full-body protection, including protective glasses, double-sided masks, isolation caps, double gloves and foot masks. To avoid infection when removing protective equipment, health care workers were not allowed to drink water, eat or go to the toilet during working hours. Some people may develop rashes and cystitis and may even become dehydrated by sweating too much [17]. Under these dangerous conditions, health care workers become mentally and physically exhausted, which can lead to many physical symptoms.

Our research had several limitations. First, this study was conducted through the self-administered questionnaire of “Wechat” program, which may lead to the deviation of self-choice. Second, the nature of the cross-sectional survey did not reflect causal relationship. Third, this study lacked follow-up data. Fourth, since this study was a comparative study, we found that there was a significant difference in working hours between SOM and non-SOM subgroups in health care workers, so it should be better to provide working hours of non-health care workers in terms of SOM and non-SOM subgroups. Unfortunately, we did not collect these data, which should be remedied in future research.

In summary, both non-medical staff and medical staff have somatization symptoms, and the prevalence rate and total score of SOM in medical staff are higher than those in non-medical staff. Factors related to severe somatic symptoms may contribute to the improvement of health policies and the formulation of prevention and treatment intervention strategies.

## Data Availability

The datasets generated during and/or analyzed during the current study are available from the corresponding author on request.

## Abbreviations

SOM: Somatization
COVID-19: The coronavirus disease 2019
WHO: the World Health Organization
PHEIC: Public Health Emergency of International Concern
PTSD: Posttraumatic stress disorder
IES-R: the Impact of Events Scale-Revised
DASS-21: Depression Anxiety Stress Scales
ISI: the Insomnia Severity Index
MINI: Mini International Neuropsychiatric Interview
OC: Obsessive-Compulsive
IS: Interpersonal Sensitivity
DEP: Depression
ANX: Anxiety
HOS: Hostility
PHOB: Phobic Anxiety
PAR: Paranoid Ideation
PSY: Psychoticism
IQRs: the interquartile ranges
BMI: Body mass index
